# The Possible Mechanisms of the Impaired Insulin Secretion in Hypothyroid Rats

**DOI:** 10.1371/journal.pone.0131198

**Published:** 2015-07-01

**Authors:** Aliashraf Godini, Asghar Ghasemi, Saleh Zahediasl

**Affiliations:** 1 Department of Physiology and Neurophysiology Research Center, Faculty of Medicine, Shahid Beheshti University of Medical Sciences, Tehran, Iran; 2 Endocrine Physiology Research Center, Research Institute for Endocrine Sciences, Shahid Beheshti University of Medical Sciences, Tehran, Iran; 3 Endocrine Research Center, Research Institute for Endocrine Sciences, Shahid Beheshti University of Medical Sciences, Tehran, Iran; University of Bremen, GERMANY

## Abstract

Although the insulin secretion deficit in hypothyroid male rats has been documented, the underling mechanisms of the effect of hypothyroidism on insulin secretion are not clear. Isolated islets of the PTU-induced hypothyroid and control rats were exposed to glibenclamide, acetylcholine, and nifedipine in the presence of glucose concentrations of 2.8 or 8.3 and 16.7 mmol/L. Glucokinase and hexokinase specific activity, glucokinase content, and glucose transporter 2 protein expression were also determined in the isolated islets. Isolated islets from the hypothyroid rats showed a defect in insulin secretion in response to high glucose. In the presence of glibenclamide or acetylcholine, the isolated islets from the hypothyroid and control rats stimulated by glucose concentration of 16.7 mmol/L secreted similar amounts of insulin. In the presence of glucose concentrations of 8.3 mmol/L and 16.7 mmol/L, nifedipine was able to diminish insulin secretion from isolated islets of both groups, indicating that probably the defect may not arise from L type calcium channels or the steps beyond depolarization or the elements involved in the acetylcoline signaling pathway. Glucokinase content and hexokinase specific activity were also the same in the control and hypothyroid groups. On the other hand, glucokinase specific activity and glucose transporter 2 protein expression were significantly (p<0.001 and p<0.01 respectively) lower in the islets isolated from the hypothyroid rats (6.50 ± 0.46 mU/min/mg protein and 0.55 ± 0.09 arbitrary unit) compared to the controls (10.93 ± 0.83 mU/min/mg protein and 0.98 ± 0.07 arbitrary unit) respectively. In conclusion, the results of this study indicated that hypothyroidism reduced insulin secretion from isolated pancreatic islets, which confirms the finding of the previous studies; in addition, the insulin secretion deficit observed in hypothyroid rats may arise from the abnormalities in some parts of the glucose sensor apparatus of the pancreatic islets including glucokinase activity and glucose transporter 2 protein expression.

## Introduction

Thyroid hormones are the critical regulators of metabolism in many cells; thus, derangement of thyroid function can affect many organs. Thyroid hormone receptor isoform alpha1 has been identified in adult pancreatic islets. It is believed that thyroid hormone is a physiological stimulus for the postnatal maturation of functional beta cells [1, 2]. Effects of hypothyroidism on insulin secretion have not been clearly elucidated. Lack of experiments on the human isolated islets insulin secretion in hypothyroidism is apparent but literature review reveals that all studies on animal isolated islets report impaired and not increased insulin secretion in response to glucose in hypothyroidism. Therefore, enhanced insulin response or concentrations that have been reported in some homeostatic models or glucose tolerance tests in hypothyroid humans or animals could attribute to insulin resistance, changes in insulin clearance, sex differences, or extent of thyroid hormones reduction, needs to be clarified. In line with other rodent studies on isolated islets [3–5], in our previous study [6], we showed impaired insulin secretion, in vivo and in vitro, in hypothyroid rats; moreover, our results showed a positive correlation between islet insulin secretion and serum T3 and T4 concentrations in thyroidectomized male rats. Nevertheless, the underlying mechanisms of the effect of hypothyroidism on insulin secretion are not yet clear.

It has also been reported that mitochondrial T3 receptor p43 regulates insulin secretion and the p43¯/¯mice have a major defect in insulin secretion and a loss of glucose-stimulated insulin secretion [7]. Glucose sensing is the initial event of glucose-stimulated insulin secretion. Therefore, it is necessary to maintain adequate expression levels of glucose transporter 2 (GLUT2) and glucokinase (GK) to ensure normal beta cell function [8]. Triiodothyronine (T3) can modulate the expression of GLUT2 protein and GK mRNA in pancreatic islets [9] and liver [10]. The effect of hypothyroidism on the expression of these glucose sensors in pancreatic islets is however not clear. The aim of this study was therefore to determine the possible mechanisms by which hypothyroidism impairs insulin secretion in rats.

## Materials and Methods

### Materials

Nifedipine, glibenclamide, acetylcholine, sodium pentobarbital, bovine serum albumin (BSA), HEPES, DL-Dithiothreitol (DTT), and 6-propyl-2-thiouracil (PTU) were purchased from Sigma (St. Louis, MO, USA); collagenase P, NADP disodium salt, ATP disodium salt, glucose-6-phosphate dehydrogenase from Roche (Roche Diagnostic, Mannheim, Germany), and all other reagent-grade chemicals from Merck (Darmstadt, Germany).

Stock solutions of nifedipine and glibenclamide, were prepared in ethanol and dimethyl sulphoxide (DMSO) and the other substances were dissolved in H_2_O or directly added into the incubation media.

### Animals and study design

Forty-two male Wistar rats, age- and weight-matched, belonging to a local stock bred in the animal facility of the Research Institute for Endocrine Sciences (RIES) of Shahid Beheshti University of Medical Sciences (Tehran, Iran) were used. The animals were randomly divided into two groups, control and PTU-induced hypothyroid (HR) rats. All animals were housed in groups of three per cage, under controlled conditions of light (12 h light/dark cycles), temperature (22 ± 3°C) with free access to food and water. The PTU-induced hypothyroid group received 0.025% (250 ppm) PTU in drinking water during the experiments [11], while the control group consumed tap water. During this period, water consumption and food intake were measured at 48-hour intervals. There was no mortality among the animals of the two groups. Avoiding suffering, the animals were anesthetized and sacrificed by heart incision prior to islet isolation. Animals were handled according to the standard principles of laboratory animal care; the study was approved by the local ethics committee of the RIES, Shahid Beheshti University of Medical Sciences. After five weeks of PTU administration, thyroid hormones were measured. The pancreatic islets were isolated and glucose induced insulin secretion in the presence of glibenclamide, acetylcholine, and nifedipine were assessed. Hexokinase and glucokinase specific activity, glucokinase content, and GLUT2 protein expression were also determined.

### Measurement of serum T3 and T4

Blood samples collected at the time of islets isolation, were centrifuged (3000×g, 10 min at 4°C) and sera were stored at -20°C for measurement of thyroid hormones. T_3_ and T_4_ were measured using ELISA kits (DiaPlus, US).

### Islet isolation

For islet isolation, the modified method of Lacy and Kostianovsky [12] with slight further modification was used. In brief, the animals were anesthetized (60 mg/kg sodium pentobarbital i.p), laparotomized, and sacrificed by heart incision. The pancreas was inflated through the bile duct with an injection of 10 ml ice-cold Hanks’ balanced salt solution (HBSS) containing 0.5 mg/mL of collagenase P, then removed, minced with scissors, and digested for 15–17 min at 37°C. Digestion was terminated by adding 30 ml ice-cold HBSS, and the tube was shaken for 1 min. The suspension was filtered through a 500 μm plastic mesh to discard any undigested tissue. After three washes with cold HBSS, islets were hand-picked under a stereomicroscope. The islets were used fresh for studies related to insulin secretion or stored frozen at -80°C until further use [6].

### Insulin secretion study

ATP-sensitive K^+^ channels and voltage-gated Ca^2+^ channels are the key components in the insulin secretion process. Pancreatic β-cells also express muscarinic acetylcholine receptors that are linked to G proteins of the Gq family. Ligand activation of these receptors facilitates glucose-induced insulin release [13]. For evaluation of insulin secretion in response to glucose and drugs, batches of 8 islets were incubated in 1 ml Krebs-Ringer solution containing: NaCl (115), KCl (5), CaCl_2_ (2.5), MgCl_2_ (1), NaHCO_3_ (24), and HEPES (16) (all in mmol/L), pH 7.4, supplemented with 5 g/L BSA and glucose concentrations of 2.8 or 8.3 and 16.7 mmol/L in the presence of glibenclamide (150 μmol/L), an ATP-sensitive K^+^ channel blocker [14], acetylcholine (100 μmol/L), a muscarinic receptor agonist [15, 16], and nifedipine (5 μmol/L), a voltage-gated Ca^2+^ channel blocker [17]. Islets were incubated for 60 min in a 37°C water bath and gassed with 95% O_2_, 5% CO_2_ for 5 min at initiation of incubation time. The incubation medium was removed and kept at -20°C for insulin measurement [11].

### Glucokinase and hexokinase activity assay

Glucose phosphorylating activity was assayed by a modified method described by Ueda et al. [18]. In brief, 300 islets were homogenized with 400 μL of lysis buffer containing: HEPES, pH = 7.4 (50), dithiothreitol (2.5) EDTA (1), KCl (100), and MgCl_2_ (5) (all in mmol/L), followed by sonication (20 KHz, 60 W, 3 × 10 s on ice). The homogenate was centrifuged at 12000×g at 4°C for 20 min and glucokinase and hexokinase activity was determined in the supernatant in the presence of 0.5 or 100 mmol/L glucose.

In the spectrophotometrical procedure, the incubation buffer contained 50 mmol/L HEPES/HCl (pH 7.4), 100 mmol/L KCl, 7.5 mmol/L MgCl_2_, 0.5 mmol/L NADP+, 0.05% BSA (w/v), 4 IU/mL glucose-6-phosphate dehydrogenase (from yeast), and glucose (0.5 or 100 mmol/L). The reaction was initiated by the addition of 5 mmol/L ATP, and the rate of reduced nicotinamide adenine dinucleotide phosphate (NADPH) production was measured at 340 nm. Correction for hexokinase activity was applied by subtracting the activity measured at 0.5 mmol/L glucose from the activity measured at 100 mmol/L glucose. Protein concentrations were determined by the Bradford method using a Bio-Rad assay kit, and enzyme activities were expressed as milli unit per milligram protein per minute (mU/mg protein/min).

### Glucokinase content

A part of the homogenate, prepared earlier for the glucokinase and hexokinase activity assay (100 μ1) was used to measure glucokinase content in pancreatic islets, using the rat glucokinase ELISA kit (Cusabio biotech, Japan, CSB-E13865r), with a sensitivity of 0.156 ng/mL.

### Western blot analysis for GLUT2

For this analysis, 250 islets were hand-picked and homogenized in 100 μL lysis buffer (150 mmol/L NaCl, 1% Triton X-100, 0.5% sodium deoxycholate, 0.1% sodium dodecyl sulphate (SDS), 50 mmol/L Tris, pH 8.0 supplemented with protease inhibitor tablet (Roche Diagnostics), followed by sonication (20 KHz, 60 W, 3 × 10 s on ice). Equal amounts of protein (60 μg) were loaded onto a 12.5% sodium dodecyl sulfate polyacrylamide gel. Following electrophoresis, the proteins were transferred overnight to polyvinylidene fluoride membranes using a constant current of 30 mA. The membranes were incubated with a 1:500 dilution of rabbit polyclonal GLUT2 antibody (Santa Cruz Biotechnology Inc., #sc-9117)) for 3 hours. The membranes were then washed three times in 0.1 M PBS/0.1% TWEEN 20 (PBST) and incubated with a secondary antibody horseradish peroxidase-conjugated goat anti-rabbit IgG (Santa Cruz Biotechnology Inc., #sc-2030)), 1:2000. Primary and secondary antibodies were diluted in the 2% nonfat dry milk in PBST. For a protein loading control, the membranes were reprobed with primary and secondary antibodies for beta actin. All incubations were performed at room temperature. The bands were scanned and transformed to digital images, and then analysed with ImageJ software.

### Statistical analysis

All data are expressed as mean ± SEM and were analyzed with GraphPad Prism software (Version 5). In normally distributed data, paired and unpaired t-tests were used to evaluate difference between two sets of data appropriately. If data was not normally distributed, Mann Whitney test was used to evaluate difference between two groups. Two-way ANOVA was used for analyzing water consumption and food intake data and Bonferroni test was used for multiple comparisons. Differences with a P value < 0.05 were considered statistically significant.

## Results

### Weight gain, water consumption and food intake

Weights of rats at the beginning of the experiment were similar in both the control (302 ± 4 g, n = 21) and hypothyroid (304 ± 7 g, n = 21) groups. As expected, weights of the control animals after 5 weeks increased (336 ± 3 g) whereas those of the hypothyroids (264 ± 6 g) decreased significantly (p<0.001).

**Fig 1:**
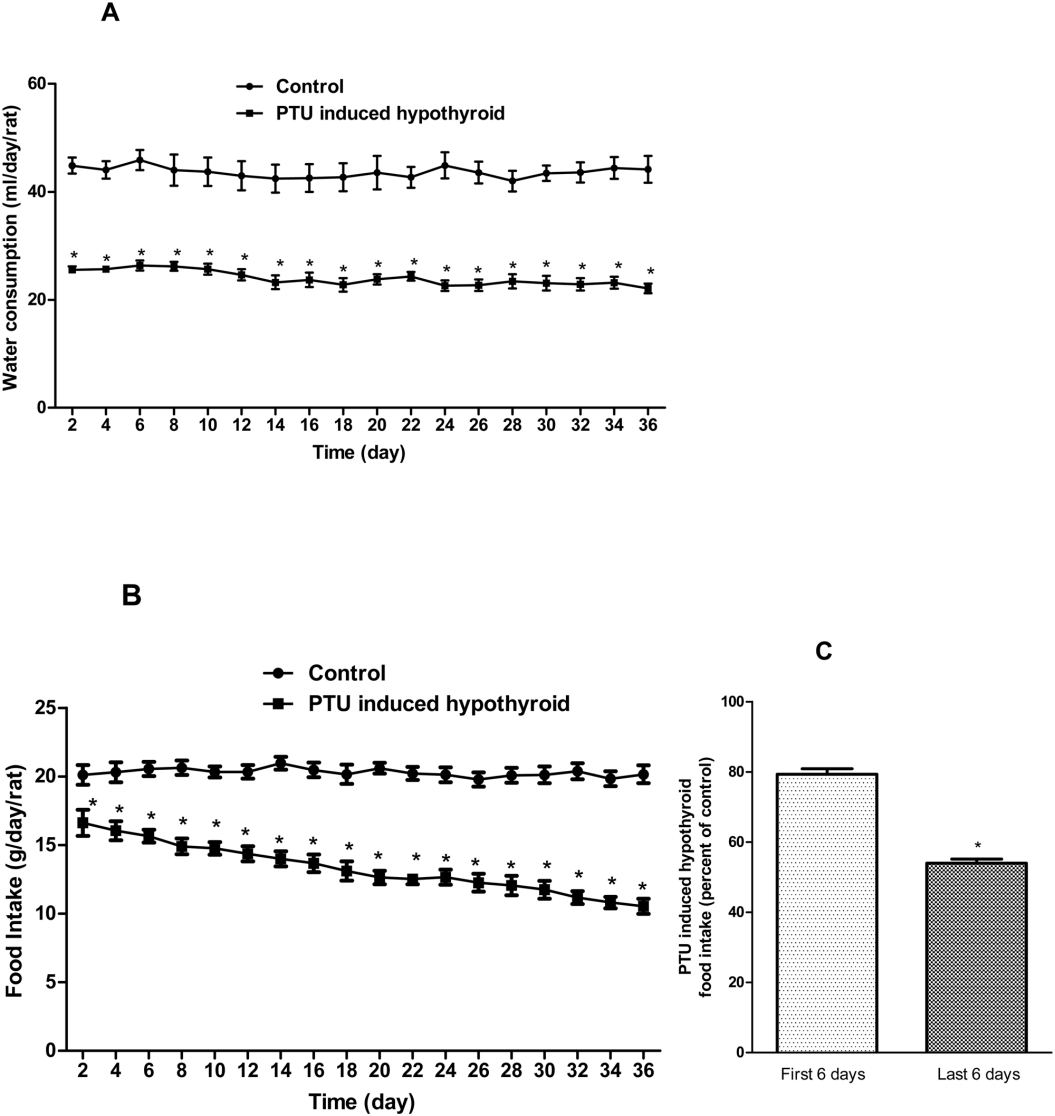
Water consumption and food intake of the animals during the experiment period. In (A) and (B), each point represents the average amounts of daily water consumption and food intake of the rats in each cage respectively (three/cage). Data are expressed as mean±SEM. A significant difference was assessed by two-way ANOVA and Bonferoni post-test. * p<0.001, represents statistically significant difference, the PTU Induced hypothyroid (7 cages) versus the controls (7 cages). In (C), each bar represents percentage of food intake in the hypothyroid rats relative to the controls. A significant difference was assessed by unpaired t test. * p<0.001, represents statistically significant difference, between the hypothyroid rats food intake in the first and in the last 6 days of the experiment period.

Details of the animals’ daily water consumption and food intake during the experiment period have been depicted in Fig 1. In order to show significant reduction of food intake in the hypothyroid rats during experiment, relative food intake of this group in the last 6 days of the experiment period was also compared with intakes in the first 6 days.

### Serum levels of thyroid hormones

Total serum T_3_ and T_4_ concentrations at the time of islets isolation were measured and results showed that serum T_3_ and T_4_ concentrations in the hypothyroid group (28.82 ± 2.34 ng/dL) and (0.33 ± 0.05 μg/dL) were significantly (p<0.001) lower than the values in the control group (67.48 ± 5.24 ng/dL and 1.97 ± 0.23 μg/dL, respectively).

### Glucose stimulated insulin secretion

**Fig 2:**
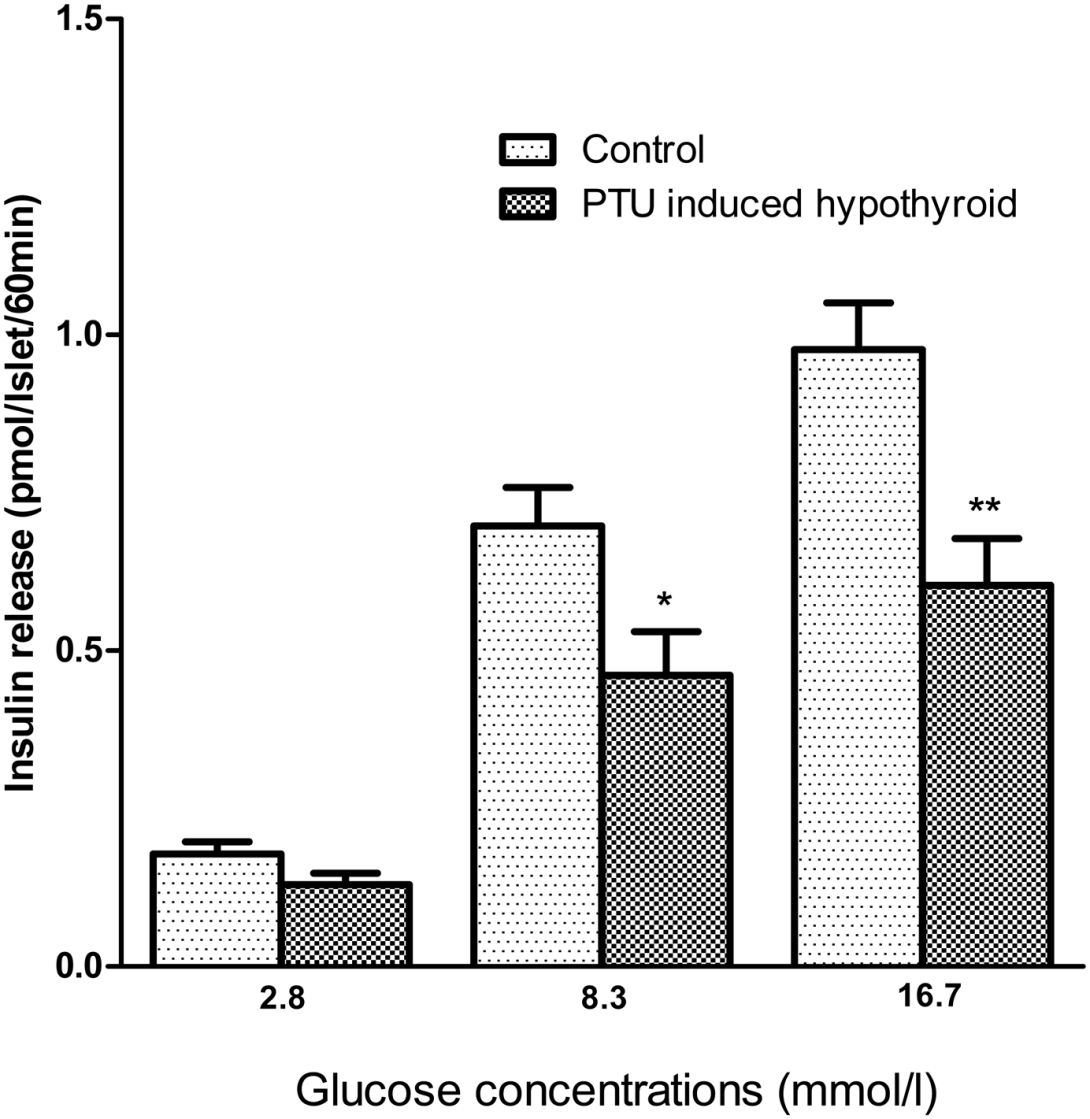
Insulin secretion by islets in different glucose concentrations. Each bar presents the mean±SEM from 12–15 batches of eight islets from 5 or 6 rats, incubated for 60 min in 1 ml of medium containing different glucose concentrations. Differences were analyzed by Mann Whitney test. *p<0.05, **p<0.01, represent statistically significant differences, the PTU induced hypothyroid rats versus the controls.

Insulin secretion in response to glucose concentrations of 2.8, 8.3, and 16.7 mmol/L in the experimental groups is given in Fig 2. In the PTU group, in response to glucose concentration of 8.3 and 16.7 mmol/L, insulin secretion was significantly lower than in the control group (P<0.05 and P<0.01 respectively).

#### Effect of glibenclamide and acetylcholine on insulin secretion

**Fig 3:**
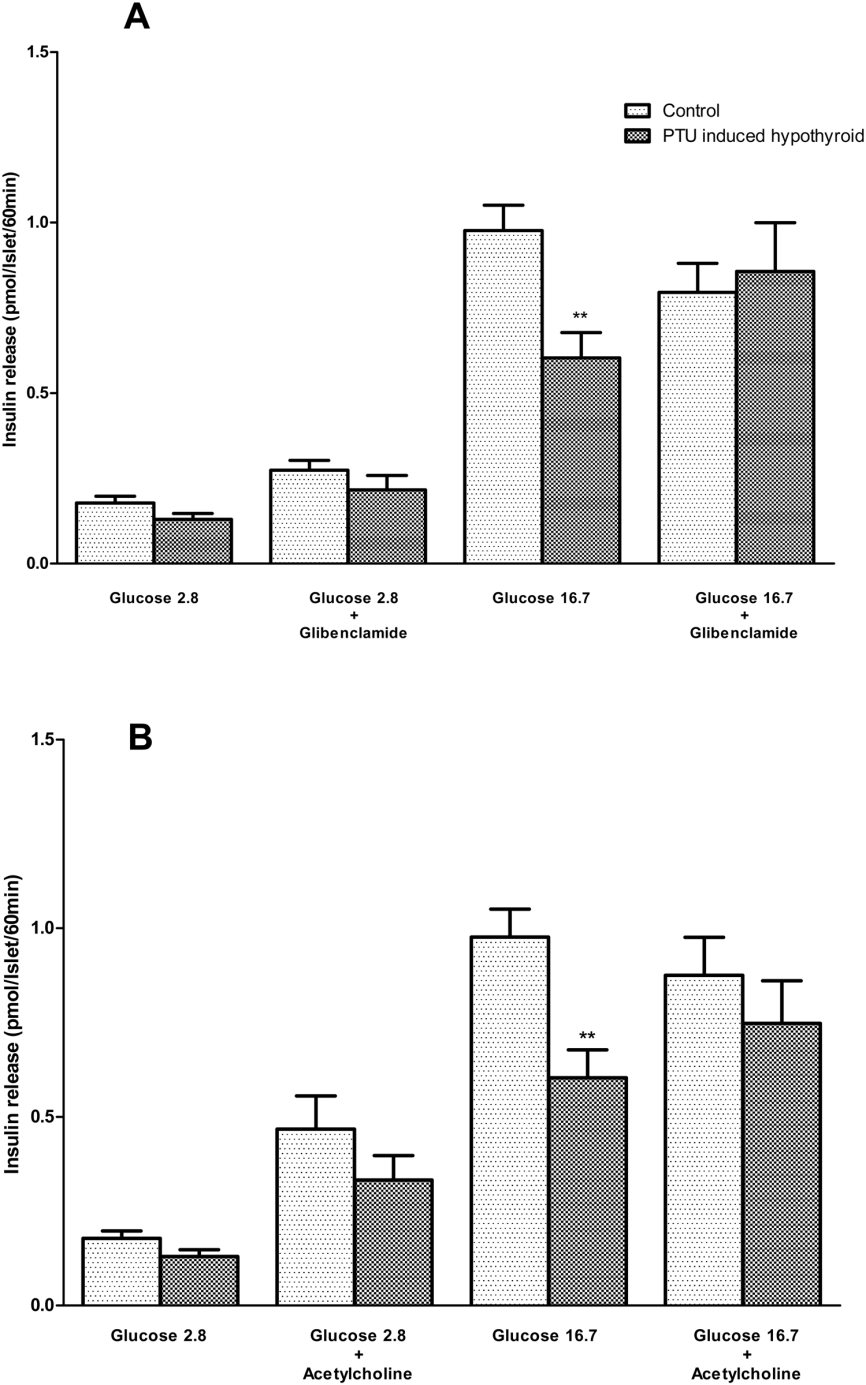
Insulin secretion by islets exposed to glucose concentration of 2.8 and 16.7 mmol/l in the presence of glibenclamide 150 μmol/L (A) and acetylcholine 100 μmol/L (B). Each bar presents the mean±SEM from 13–15 batches of eight islets from 5 or 6 rats, incubated for 60 min in 1 ml of medium containing the glucose concentrations and secretagogues. Differences were analyzed by Mann Whitney test. **p<0.01, represents statistically significant difference, the PTU induced hypothyroid rats versus the controls.

In the presence of glibenclamide (part A) or acetylcholine (part B), the isolated islets from the hypothyroid and control rats stimulated by glucose concentration of 16.7 mmol/L secreted similar amounts of insulin (Fig 3).

#### Effect of nifedipine on insulin secretion

**Fig 4:**
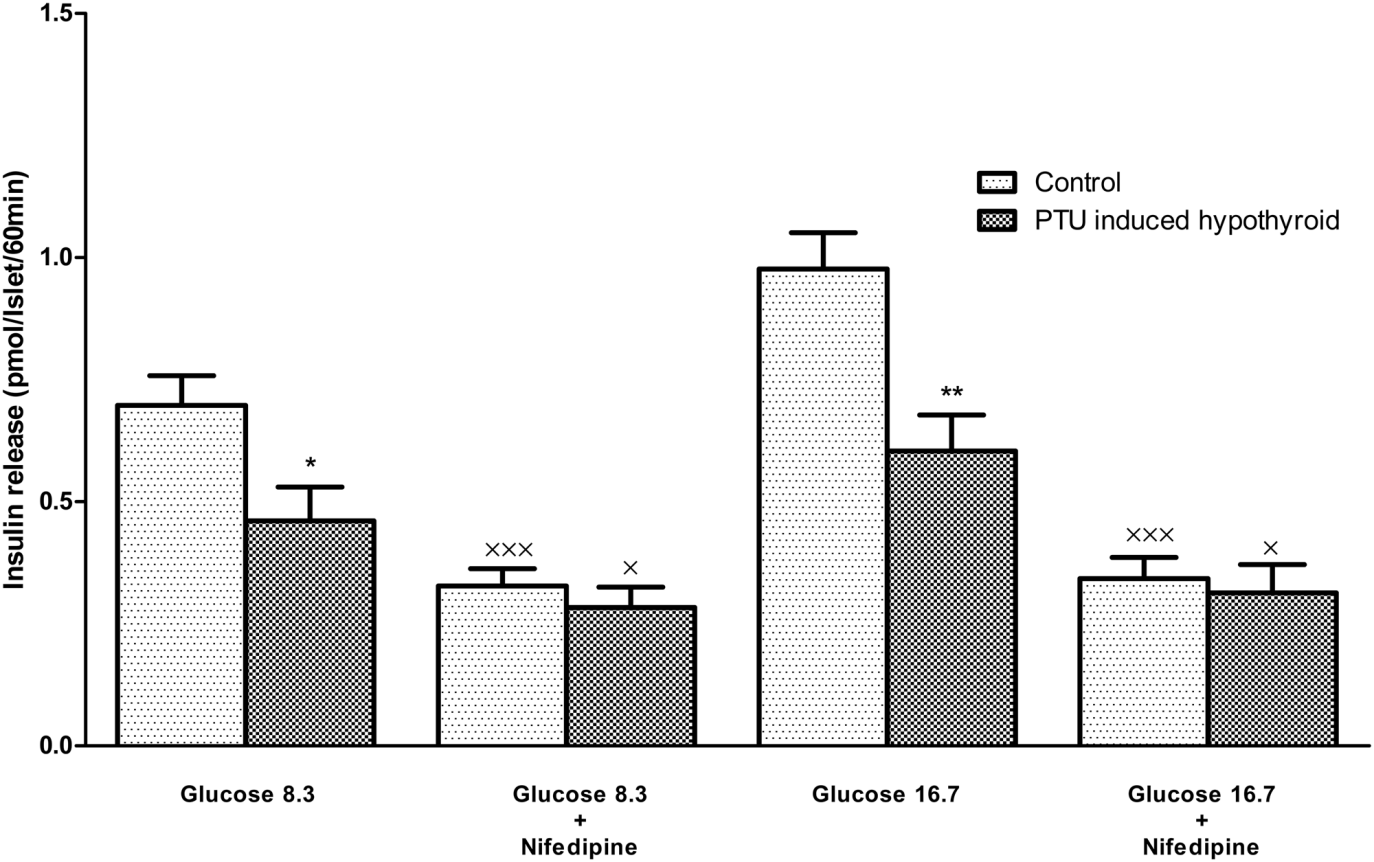
Insulin secretion by islets exposed to glucose concentration 8.3 and 16.7 mmol/L in the presence of nifedipine 5 μmol/L. Each bar presents the mean±SEM from 12–17 batches of eight islets from 5 or 6 rats, incubated for 60 min in 1 ml of medium containing the glucose concentrations and nifedipine. Differences were analyzed by Mann Whitney test. *p<0.05, **p<0.01 represent statistically significant differences, the PTU induced hypothyroid rats versus the controls. ×*p*<0.05, ×××*p*<0.001 represent statistically significant differences, in the presence of nifedipine compared to its absence in each group.

Results showed that nifedipine could diminish insulin secretion significantly in response to glucose concentrations of 8.3 and 16.7 mmol/L in both groups (Fig 4).

### Hexokinase and glucokinase specific activity and glucokinase content

**Fig 5:**
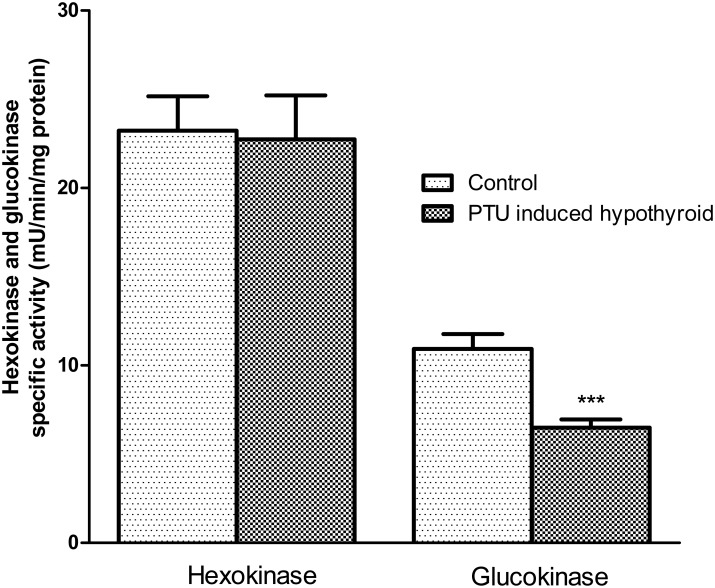
Hexokinase and glucokinase specific activity. Each bar presents the mean±SEM from 9 cups of 9 rats. Each cup contained 300 islets in 400 μL of lysis buffer. Differences were analyzed by unpaired t test. ***p<0.001, represents statistically significant differences, the PTU induced hypothyroid rats versus the controls.

There was no significant difference between specific activity of enzyme hexokinase, assayed in the isolated islets of the PTU-induced hypothyroid and the control groups. Nevertheless glucokinase specific activity of the islets isolated from the PTU-induced hypothyroid was significantly (p<0.001) lower than that of the controls (Fig 5). The glucokinase content was the same in the islets isolated from the control (138.2 ± 3.7 ng/mg of protein, n = 9) and PTU induced hypothyroid (158.3 ± 12.7 ng/mg of protein, n = 9) groups.

### GLUT2 protein expression

**Fig 6:**
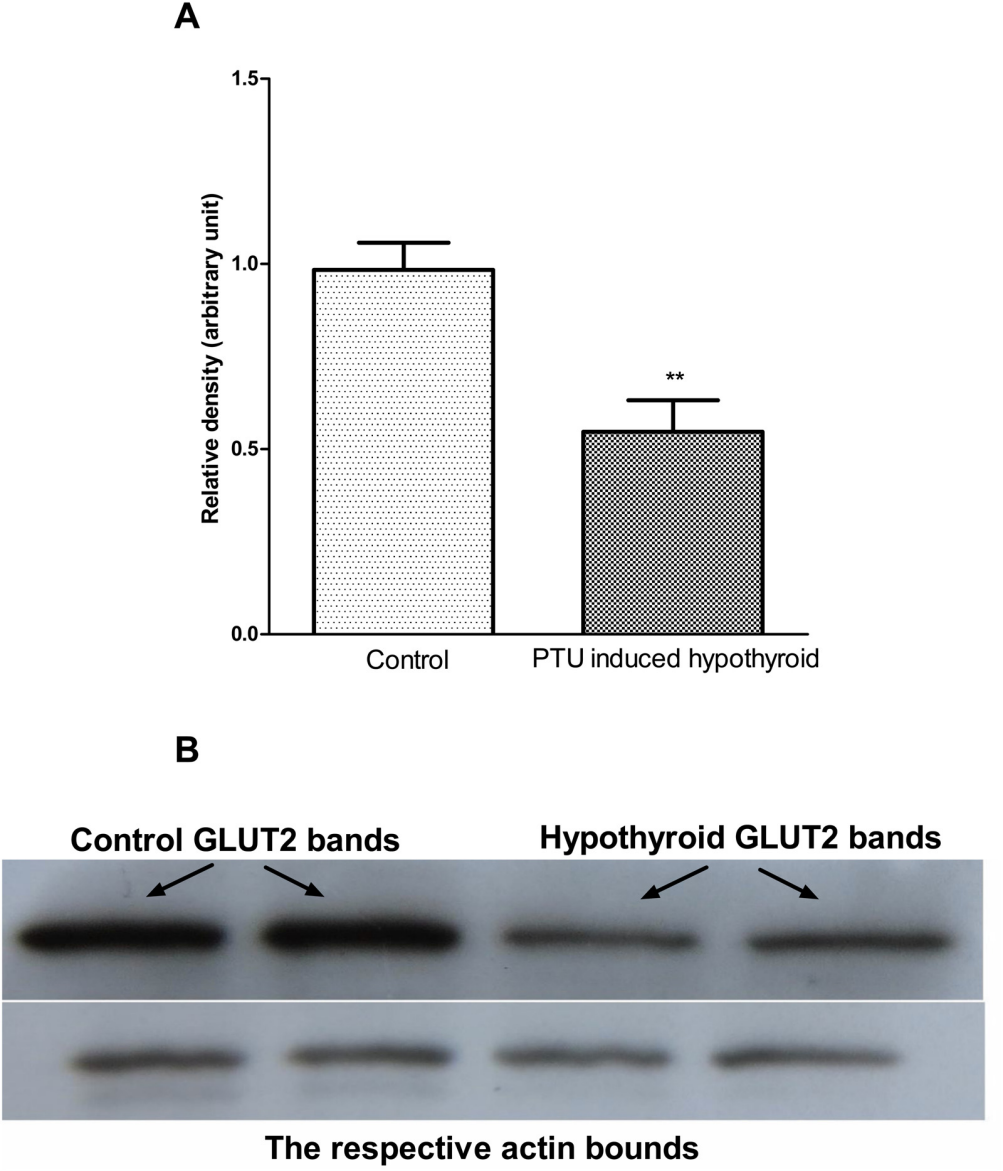
Western blot analysis of GLUT2 protein in isolated islets in hypothyroidism. Data are expressed as mean±SEM from 10 protein bands of 5 rats in each group. Unpaired t test. **p<0.01, represents statistically significant difference, the PTU induced hypothyroid rats versus the controls.

Western blot results showed that the amount of GLUT2 protein expression in islets isolated from the hypothyroid rats was significantly (p<0.01) lower than the values in the controls (Fig 6).

## Discussion

Similar to previous data, results of this study confirm that hypothyroidism reduces glucose stimulated insulin secretion in rats, a finding that may be due to abnormality in some parts of glucose sensor apparatus of the beta cells, including GLUT2 protein levels and glucokinase specific activity. Our results also show that observed decreased insulin secretion may not be related to L type calcium channels, the steps beyond depolarization, the elements involved in the acetylcholine-signaling pathway, or changes in hexokinase activity or glucokinase content.

In our previous study [6], in addition to the impaired insulin secretion, in vivo and in vitro, in hypothyroidism, a positive correlation was demonstrated between islet insulin secretion and serum T_3_ and T_4_ concentrations in thyroidectomized male rats; we previously discussed that the controversy about insulin secretion in hypothyroidism may be related to the gender of animals used in some experiments, presence or absence of insulin resistance, or the severity of the hypothyroidism. Tonooka and Kobayashi, have reported the circadian variation of plasma TSH and a sex related variation in the acute stage of hypothyroidism resulting from PTU treatment; they showed that following administration of PTU, nycthemeral fluctuation in male rats rapidly disappeared, whereas it was preserved in female rats. Furthermore, female rats showed higher concentrations of plasma TSH after PTU treatment compared to male ones [19]. However a part of the controversy in addition to sex differences, might arise from higher plasma insulin concentrations which may develop as a compensation in insulin resistance [20]. It is worth mentioning that enhanced insulin response is only observed in some homeostatic models or glucose tolerance tests while all studies in isolated islets including our current one have documented impaired and not increased insulin secretion in hypothyroidism.

The insulin content of islets in our previous study was also similar in the control and hypothyroid groups, suggesting that the reduced insulin release was not attributable to reduced available pool of insulin, but rather to alterations in the insulin secretion pathways [6].

Glucose is transported by membrane-bound GLUT2 into the beta cell, where it is phosphorylated by GK to yield glucose 6-phosphate, thereby initiating glycolysis. The resultant increase in the ATP/ADP ratio causes the closure of the ATP-sensitive K^+^ channels (K_ATP_ channel) and subsequent depolarization of the plasma membrane. Oscillatory changes in the membrane potential activates the opening and subsequent closure of the voltage-gated Ca^2+^ channels to allow an influx of extracellular Ca^2+^ [21] and an increase in cytoplasmic Ca^2+^ triggers insulin exocytosis [22, 23]. Shimoni and Light have shown that T_3_ regulates several K^+^ currents in the rat heart and K^+^-ATP channels are under the influence of long-term regulatory effect of thyroid hormone [24, 25]. They have reported that hypothyroidism could significantly decrease the sensitivity of K^+^-ATP channels to ATP [25].

It has also been indicated that muscarinic stimulation of pancreatic beta cells leads to a series of biochemical events that are usually associated with the activation of Gq-type G proteins, including the activation of phospholipase C, protein kinase C, and phospholipase A2. Stimulation of these signaling cascades eventually results in elevated intracellular calcium levels and an increase in the efficiency of calcium-dependent exocytosis of insulin-containing storage vesicles [26–28]. M3 receptor activity in beta cells is critical for the maintenance of blood glucose homeostasis and disruption of this pathway in transgenic mice results in reduced insulin release and impaired glucose tolerance. In contrast, increasing the number of beta-cell M3 receptors in transgenic mice leads to enhanced insulin release and greatly improved glucose tolerance [29, 30]. Nevertheless in the present study, there was no difference between insulin secreted in response to high glucose stimulation in the presence of glibenclamide or acetylcholine in isolated pancreatic islets. It may indicate that the pathways recruited by these secretagogues are probably not involved in the insulin secretion deficit.

In the present study, we used a Ca^2+^ channel blocker to investigate the involvement of calcium channels in the altered islets insulin secretion of hypothyroid rats. Yu et al, have shown that thyroid hormones increase L-type calcium channel mRNA expression and L-type calcium current in the rabbit myocytes [31]. It has been demonstrated that in the sinus node of the left atrium, some of the ion channels, such as L-type calcium channels are targets of thyroid hormone action [32]. In our study, in both groups, nifedipine significantly suppressed islet insulin secretion in the presence of 8.3 and 16.7 mmol/L glucose concentrations, indicating thereby that L-type calcium channels are still responsive to blockers in pancreatic islets and probably they are not responsible for the insulin secretion deficit observed in hypothyroidism.

Impairment in glucose sensing contributes to pancreatic beta cell dysfunction. The GK and GLUT2 are key molecules with a high Km for glucose phosphorylation and glucose transport respectively, which affect various processes of glucose sensing in pancreatic beta cells [33].

At the entry of glycolysis, GK plays a primary regulatory role in the control of glucose metabolism in beta cells [34–36]. Two important properties enable GK to function as a glucose sensor in beta cells, distinguishing it from other hexokinases. The first property is its relatively lower affinity for glucose than other hexokinase isoforms. The Km of glucokinase is 6 mmol/L, placing it in the middle of the normal blood glucose range (4–10 mmol/L), while other hexokinases function at maximal velocity at this glucose concentration. The second property is that it is not inhibited by its product, often a regulatory feature in metabolism, which enables its continued activity in spite of a high glycolysis load. GK is thus the rate-limiting step in beta cell glucose metabolism and is considered to be an important glucose sensor [37].

Our results showed that specific activity of GK but not hexokinase was significantly lower in hypothyroid islets compared to that of controls; this reduction of activity is not related to reduction of GK content because it was not changed significantly but may reflect changes in its function. Studies have shown that thyroid hormones can modulate the activities of many enzymes including pancreatic and hepatic GK [9, 38]. It appears that reduced GK activity leads to lowering glycolytic flux, reducing the ATP/ADP ratio, decreasing numbers of closed ATP-sensitive K channels and reducing insulin secretion. Data supports the notion that, under certain conditions, decreased activity of glucose-phosphorylating enzymes could contribute to insulin hyposecretion. It can be suggested that, hypothyroid rats present alterations in the regulation of glucose-induced insulin secretion, at least partly as a result of changes in early stages of the glycolytic pathway.

In our study, GLUT2 protein level in the isolated islets was significantly reduced in hypothyroid rats. Whereas some reports suggests that glucose stimulated insulin secretion could proceed normally even in the presence of low levels of this transporter [39], others support a specific role for GLUT2 [40, 41]. It has been found that GLUT2 plays an essential role in glucose sensing in different tissues [42–45]. GLUT2 null mice are hyperglycemic, hypoinsulinemic, hyperglucagonemic, and glycosuric and die within the first 3 weeks of life [46, 47]. Reexpressing GLUT2 in beta cells of GLUT2 null mice results in nearly normal glucose stimulated insulin secretion and fatality [48]. Studies with gene knockout mice have indicated that GLUT2 is also required for the function of glucose sensors present in the hepatoportal vein area and in the central nervous system [49]. It has been shown that glucokinase activity can be elevated in the presence of increased GLUT2 protein levels [50]. It has also been indicated that GLUT2 protein and mRNA levels decrease in the mouse liver in hypothyroidism and increase in hyperthyroidism [10, 51]. To the best of our knowledge, this is the first study to evaluate pancreatic islets glucokinase activity and protein and GLUT2 protein in hypothyroidism.

Thyroid hormone receptor isoform alpha1 has been identified in adult pancreatic islets. It is believed that thyroid hormone is a physiological stimulus for the postnatal maturation of functional beta cells [1]. Data shows that overexpression of MafA in neonatal islets to approximately adult MafA mRNA levels induces glucose-responsive insulin secretion and thus facilitate the functional maturation of beta cells [52]. Mazzucato et al showed that in vitro exposure of immature islets to T_3_ enhances MafA expression and increases glucose responsiveness, effects that are abolished in the presence of dominant negative MafA [53]. Data demonstrate that MafA-deficient mice and islets are unable to respond to glucose. It has been shown that Pdx1 expression is regulated by MafA in beta cells and is diminished in MafA-deficient mice [54, 55]. Pdx1¯/¯ islets display abnormal response to glucose and KCl accompanied with decreased protein levels of Glut-2 and glucokinase [56]. Pdx1 and MafA display beta cell restricted expression [57–59]. Therefore, it needs to be clarified whether the effect of hypothyroidism on glucokinase activity and GLUT2 expression in the pancreatic islets, observed in this study, is due to the general regulating effect of thyroid hormones, because it also takes place in the liver, or whether is specifically through Pdx1 and MafA.

In conclusion, the results of this study indicate that hypothyroidism reduces insulin secretion from isolated pancreatic islets, confirming the results of previous studies; in addition, the reduction observed in insulin secretion from pancreatic islets of hypothyroid rats may be due to decline in both levels of GLUT2 protein and GK functioning.
